# Alternating exosomes and their mimetics as an emergent strategy for targeted cancer therapy

**DOI:** 10.3389/fmolb.2022.939050

**Published:** 2022-08-10

**Authors:** Lokesh Chandra Mishra, Utkarsh Pandey, Abhikarsh Gupta, Jyotsna Gupta, Monal Sharma, Gauri Mishra

**Affiliations:** ^1^ Department of Zoology, Hansraj College, University of Delhi, New Delhi, India; ^2^ Department of Zoology, Swami Shraddhanand College, University of Delhi, New Delhi, India; ^3^ Department of Microbiology, Swami Shraddhanand College, University of Delhi, New Delhi, India; ^4^ Betterhumans Inc., Gainesville, FL, United States; ^5^ Division Radiopharmaceuticals and Radiation Biology, Institute of Nuclear Medicine and Allied Sciences, New Delhi, India

**Keywords:** exosome, targeted delivery, exosome mimetics, biomarker, therapeutics

## Abstract

Exosomes, a subtype of the class of extracellular vesicles and nano-sized particles, have a specific membrane structure that makes them an alternative proposition to combat with cancer through slight modification. As constituents of all most all the primary body fluids, exosomes establish the status of intercellular communication. Exosomes have specific proteins/mRNAs and miRNAs which serve as biomarkers, imparting a prognostic tool in clinical and disease pathologies. They have efficient intrinsic targeting potential and efficacy. Engineered exosomes are employed to deliver therapeutic cargos to the targeted tumor cell or the recipient. Exosomes from cancer cells bring about changes in fibroblast via TGFβ/Smad pathway, augmenting the tumor growth. These extracellular vesicles are multidimensional in terms of the functions that they perform. We herein discuss the uptake and biogenesis of exosomes, their role in various facets of cancer studies, cell-to-cell communication and modification for therapeutic and diagnostic use.

## Introduction

Exosomes are recognized as a subtype of the class of Extracellular vesicles (EVs). These nano-sized particles appear as small, flattened hemispheres with a diameter of 40–150 nm and a density of 1.13–1.21 g/ml (Kalluri, 2016; Gilligan and Dwyer, 2017; Kalimuthu et al., 2018; You et al., 2018; Głuszko et al., 2019; Zhao and Xie, 2019). The orientation of the surrounding lipid bilayer membrane can be regarded unique as it serves as a reflection of the intrinsic cell from which the exosomes have originated (Kalluri, 2016; Kalimuthu et al., 2018; Głuszko et al., 2019; Zhao and Xie, 2019). The structural specificity of the membrane confers properties, proving to be useful in cancer treatments through selection of preferably modified exosomes (You et al., 2018).

Exosomes are remarkable constituents of all the major body fluids, including plasma, saliva, urine, cerebrospinal fluid (Zhao and Xie, 2019) and are also present as secretions or discharges of cells such as red blood cells, platelets, lymphocytes, dendritic cells and cancer cells (Głuszko et al., 2019). Nucleic acids like RNA [mRNAs, microRNAs (miRNA) and long noncoding RNA (IncRNA)] (Głuszko et al., 2019; Zhao and Xie, 2019), cellular proteins and lipids represent the contents of exosomes (Kalluri, 2016; Gilligan and Dwyer, 2017; Kalimuthu et al., 2018; You et al., 2018). Noteworthy, the presence of DNA in the exosomes is considered rather contradictory. It is thought to share a relation with the source of the exosomes in consideration (Fais et al., 2013). These contents are protected from degradation and are taken up *via* fusion by the target cell acting as receiver, thus establishing the status of exosomes as means of intercellular communication (Kalluri, 2016; You et al., 2018; Głuszko et al., 2019).

Majority of exosomal proteins are universal and are referred to as “exosome markers” (Głuszko et al., 2019). These include TSG101, ALIX and ESCRT complex (exosome biogenesis), RabGTPases and annexins (exosome delivery and membrane fusion), heat shock proteins (HSP70, HSP90), integrins, tetraspanins (CD9, CD63, CD81 and CD82), MHC class II proteins, epithelial cell adhesion molecules (EpCAM) and members of the human epidermal receptor (HER) family (Fais et al., 2013; Kalluri, 2016; Głuszko et al., 2019). Apart from the signature molecules present within them, there are certain specific proteins and nucleic acids acquired by the exosomes from their native cell types, which can serve as biomarkers for the identification of various diseases including cancer (Fais et al., 2013). Numerous genes and proteins have been identified in lung cancer cells and tissues that can serve as exosomal biomarkers for lung cancer. ZEB1, TRAF4, and TGF-β1 are involved in lung cancer metastasis by EMT proteins while PD-L1, EGFR, TLR7 and TLR8 are involved in inhibiting the immune system. Like other cancers, exosomes derived from breast cancer cells are enriched with certain miNAs that are not abundant in healthy cells. miR-372, miR-101 and miR-373 were not found in significantly higher proportions in exosomes from breast cancer cells. Certain nucleic acid molecules and proteins may also serve as diagnostic biomarkers of colorectal cancer (CRC). Among miRNAs, around 7 to 11 molecules have been identified to be differentially expressed in CRC patients. Similarly, in colorectal Cancer, the cell surface proteoglycan Glyptican1 (GPC1) serves as the most prominent biomarker of pancreatic cancer.

**FIGURE 1:**
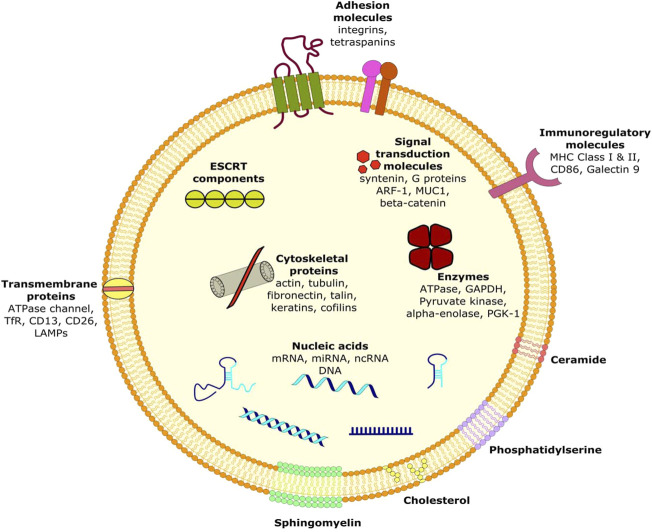
Ultrastructure of exosome showing its composition.

Ovarian cancer cells derived exosomes include membrane proteins, Rab proteins, annexin proteins, tetraspanins, heat shock proteins *etc*. can be used to potentially identify the malignancy early in its development. Besides, *Helicobacter pylori* infection is the most common factor that predisposes a person to develop gastric cancer by transporting the virulence factor CagA (mediator of gastric disorders) to epithelial cells and mesenchymal-epithelial transition factor (MET) protein to macrophages. The exosomes are enriched in lipids such as cholesterol, sphingomyelin, hexosylceramides, phosphatidylserine, phosphatidylcholine, phosphatidylethanolamines and saturated fatty acids (Figure 1) (Fais et al., 2013; Kalluri, 2016). Moreover, presence of lipid-raft like domains (due to membrane associated lipid-raft proteins) and/or phospholipid scramblase (responsible for translocating phospholipids of membrane leaflets) have also been consistently reported (Fais et al., 2013).

Exosomes are found to be enriched with the presence numerous classes of RNAs encompassing various expressed and significantly matured miRNAs and mRNAs. They are known to have eminent effects on physiological and developmental aspects of growth, development and regulation of expression in the recipient cell (Kalluri, 2016; Głuszko et al., 2019). Interestingly, recent studies have established a strong notion governing the fact that exosomes are acting as molecular vehicles by showing the presence of placental specific miRNA in the maternal blood exported *via* mature trophoblast, imparting the ability to modify genetic expressions (Fais et al., 2013).

### Exosomes: Biogenesis

Exosomes are constitutively produced by cells through the inward budding of the plasma membrane leading to the formation of intracellular endosomes, which further fold in to form multivesicular bodies (MVBs). These MVBs contain nano-sized vesicles which after fusing with the plasma membrane release their contents to the extracellular space leading to the release of exosomes (Fais et al., 2013; Kalluri, 2016).

**FIGURE 2:**
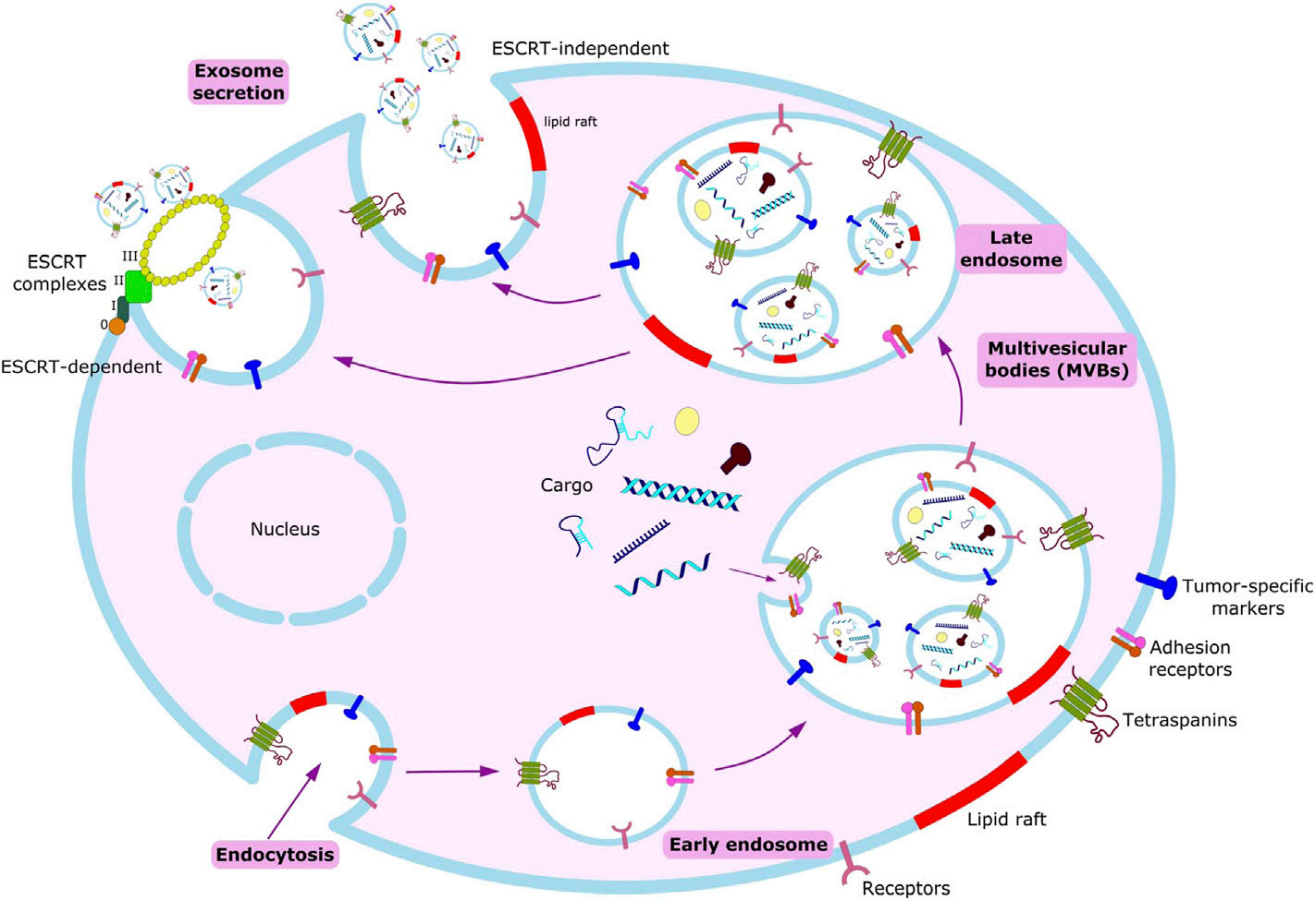
Scheme of exosome biogenesis and secretion.

Exosomes are predominantly generated by the aid of endosomal sorting complexes required for transport complexes (ESCRT complexes) (Figure 2). ESCRT is composed of four complexes namely ESCRT-0, ESCRT-I, ESCRT-II and ESCRT-III along with several associated accessory proteins (ALIX, VPS4, Tsg101, VTA1) which sort ubiquitinated cargo proteins on the inner leaflet of the endosomal membrane and caused subsequent scission thereby releasing the exosomes (Kalluri, 2016; Yue et al., 2020).

Biogenesis begins with endocytosis, enclosing bioactive molecules, forming endosomes. Endosomal membrane further undergoes inward budding, enclosing the molecules and forming multivesicular bodies (MVBs). Exosomes form from these MVBs, either through ESCRT-dependent or ESCRT-independent pathways.

Based on recent studies, a key system has been elucidated called the Syndecan-syntenin pathway which is responsible for controlling the generation of endosomal vesicles that release exosomes as well as delivering cargo inside these vesicles. Syntenin bound to syndecans with the help of extracellular heparansulphate chains communicate with a myriad of signaling and adhesion moieties including ALIX protein which links the Syndecan-syntenin pathway with the ESCRT machinery (Fares et al., 2017; Hessvik and Llorente, 2018).

Remarkably, numerous studies have also indicated the presence of an ESCRT-independent pathway for exosome biogenesis and loading which is mediated by lipids and associated proteins. Proteins such as A2, RAB5/7/27, TSG101 have been identified to play important roles in exosome biogenesis (Kalluri, 2016). Several transmembrane proteins of tetraspanin family such as CD9, CD63, CD82, Tspan8 are also involved in ESCR-independent exosome generation. Another protein namely small integral membrane protein of the lysosome/late endosome (SIMPLE) has also been shown to positively influence the release of exosomes. Apart from proteins, various lipids such as Phosphatidic acid, ceramide, glycosphingolipids, lyso-phospholipid are also inducers of exosomebiogenesis (Hessvik and Llorente, 2018; Yue et al., 2020).

ESCRT-dependent or ESCRT-independent mechanisms are not entirely exclusive. Exosome biogenesis is instead a coordinated and synergistic outcome of these mechanisms wherein their presence or absence in a particular cell type and/or cellular homeostasis manipulates intraluminal vesicle number and size in addition to cargo sorting and loading (Yue et al., 2020). Moreover, exosome secretion is eminently regulated by the conditions of the cellular microenvironment. In tumor cells, stressful conditions arise due to several factors such as chemotherapeutics, irradiation, starvation and most notably hypoxia which together lead to increased production of exosomes (Głuszko et al., 2019).

### Exosomes uptake

Exosomes from the extracellular space can adhere to the cell in its proximity nonspecifically (Tian et al., 2013) or be attached *via* a specific ligand-receptor complex (Ohno et al., 2013). They can either exert their functional effects by direct activation of a signaling pathway (Al-Nedawi et al., 2008; Cossetti et al., 2014; Patel et al., 2016) or be internalized to transfer their cargo inside the cell, *via* endosomal maturation: the endosomal-lysosomal degradative pathway (Nakase and Futaki, 2015). Non-specific internalization of exosomes has been shown to occur in both normal and transformed cell lines (Svensson et al., 2013). The uptake depends on the recipient cell and not on the origin of the exosomes (Horibe et al., 2018). It is an energy dependent process, as evidenced by attenuation of the uptake when incubated at 4°C or with compounds interfering with cell function (Morelli et al., 2004). It has been shown that low pH conditions, a consequence of hypoxia in the cell interstitium and a characteristic of tumor microenvironment lead to rupture of the exosomal membrane and subsequent uptake of its cargo through macropinocytosis (Joseph et al., 1996; Gatenby and Gillies, 2004; Taraboletti et al., 2006; Parolini et al., 2009). The two most frequently reported ways of exosomal uptake by cancer cells are Lipid-raft mediated endocytosis and macropinocytosis.

**Clathrin-independent endocytosis (CIE)** is reported to occur through membrane proteins, CAV-1, flotillin-1 and RhoA that are known to be components of lipid-rafts: the microdomains of plasma membrane that contain high cholesterol and glycosphingolipid concentration, involved in endocytosis (Costa Verdera et al., 2017). Local disruption of actin network and inhibition of dynamin recruitment to plasma membrane: both of which are important in CIE, decreased exosomal uptake, suggesting its role in endocytosis (Tian T. et al., 2014). Although, in a study, CAV1 knock-out cells showed increased uptake of exosomes, in most of them, CAV-1 has been shown to play important role in CIE, as described above (Feng et al., 2010; Chaudhary et al., 2014).

**Clathrin-dependent endocytosis (CDE)** although shown to be taking part in uptake in some cancer cells, is majorly non-existent (Tian T. et al., 2014).

**Macropinocytosis (MP)** is another widely discussed pathway of exosomal uptake by cancer cells. Exosomes can themselves induce MP, thus facilitating their uptake (Yan-Liang et al., 2014). EGFR expression has been shown in many tumors, when activated by EFG, enhanced uptake through MP *via* activation of Rac, leading to cytoskeletal organization and subsequent induction of MP (Nakase et al., 2015).

## Role of exosomes in mitigating cancer metastasis

Exosomes and their contents function together as a unit, facilitating the promotion of malignancy and tumorigenic effects, aiding the ability of native epithelial cells. Serving as an alternative to conventional cell-based therapies, exosomes are currently being engineered to deliver therapeutic cargos to the targeted recipient or tumor cells (Gilligan and Dwyer, (2017); You et al., 2018; Głuszko et al., 2019). Furthermore, the most presumptuous property of cancer i.e. metastasis is governed by the localized impression of exosome-mediated signaling. The influence is diverse and can be either due to site-production of exosomes or through uptake by a distant recipient cell (Kalluri, 2016). The mediators proffered between tumor and their microenvironments are under constant modulation and are known to play a key role in cancer immunotherapy. Tumor-derived exosomes (TEXs) are one such modification, suitable for imparting chemotherapeutic resistance, influenced by various other strategies, survival time and tumor growth (You et al., 2018).

A study in 2018 accorded for observing induced apoptosis along with a combined outcome of reduced metastasis and prolonged survival when exosomal cargo, siKras^G12D-1^, in the donor cells of Bone marrow MSCs-derived exosomes (BM-MSC), was electroporated with pancreatic cancer as the tumor model (Melo et al., 2014; Mendt et al., 2018; You et al., 2018). The most talked about are the cancer cell-derived exosomes that have been found accountable for the transformation of benign epithelial cells into malignant cells (Kalluri, 2016). Melo SA *et al.* demonstrated how cancer exosomes differ from normal exosomes as the former possesses the capability of independent transcription of miRNA through a Dicer-dependent pathway (Melo et al., 2014; Kalluri, 2016). It was reported that breast cancer associated exosomes enriched in miRNA exhibit the presence of RISC-Loading Complex (RLC), thus efficiently mediating the process of silencing and miRNA biogenesis (Mendt et al., 2018).

Additionally, a research study in 2015 investigated the chemo-sensitive resistance in tumors displayed by patients with Hepatocellular carcinoma (HCC). A cumulative effect of metastasis, inadequate prognosis and loss of miR-122 encapsulated within exosomes caused the patients to develop a resistance to chemotherapies involving agents such as 5-fluorouracil (5-FU) and doxorubicin (Lou et al., 2015). A subsequent observation was made, while testing, whether a modification revolving around Adipose-derived MSCs (AMSCs) can prove helpful in restoring the lost chemosenstivity *via* expression of miR-122. Reduction in the tumor mass and volume was observed when the engineered exosomes administered intra-tumoral to BALB/c nude mice with HepG2 tumors, combined with sorafenib treatment, thus increasing the HCC cell sensitivity (Lou et al., 2015; Gilligan and Dwyer, 2017).

Likewise, Tumor-derived exosomes (TEXs) regulate the process of tumor formation due to release of immunosuppressive molecules such as Fas-ligand (FasL), the expression of which contributes to resistance and malignant niche selection. Expression of exosomal secretions can also be traced to the responses and correlated levels of Tumor necrosis factor-related apoptosis-inducing ligand (TRAIL), interleukin 10 (IL-10), programmed death-ligand 1 (PD-L1), neo-angiogenesis factors and several other microenvironment conditioning factors, e.g., transforming growth factor β1 (TGF-β1), prostaglandin E2 (PGE2) and ecto-enzymes engaged in the adenosine pathway (CD39 and CD73) (Głuszko et al., 2019).

The current cancer therapeutics deal with targeted destruction of both cancer stem cells (CSCs) alias cancer-initiating cells (CICs) as well as the non-CSCs. Several investigative experiments suggest that the CSC population is not static, and can be effectively reconfigured. The transformation of non-CSCs to CSCs results in regaining or acquiring stemness phenotype in the non-CSC tumor microenvironment which can be achieved by CSC-derived exosomes (Lin et al., 2013; Hu et al., 2015; Donnarumma et al., 2017).

The progressive inter-conversion establishes an equilibrium between the exosomal derived CSCs and non-CSCs, involving various cellular signaling pathways, bio-active cell cargo, molecular sorting and transport. Thus, a prospective scheme can be developed concerning possible mechanisms like cancer initiation, progression, metastasis, relapsing and resistance to therapies by modifying exosome contents in the cancer surrounding. A controlled and regulated interaction between CSCs and non-CSCs can be accomplished, which will prove to be a more beneficial and novel therapeutic strategy (Sun B. et al., 2018).

### Role of exosomes in cancer: Signaling

Exosomes are capable of acting as transporters for different molecules aided by several types of signaling mechanisms and pathways that can operate over varying distances (Whiteside, 2017). Significantly, they play important roles in maintaining cancer-related functions such as metastasis, angiogenesis and regulating tumor micro-environment (Rachel et al., 2017; Whiteside, 2017; Sun Z. et al., 2018). Exosomes generated from Cancer Stem Cells (CSCs) are believed to induce stemness in non-CSCs thereby maintaining a state of equilibria in the tumor-microenvironment (Sun Z. et al., 2018). The ability of exosomes to act as communication vehicles is due to the presence of diverse signaling molecules on their surface derived from their parent cell as well as the presence of various nucleic acids, enzymes and factors inside them, thereby making them capable of acting as efficient mediators of cancer metabolism (Whiteside, 2017). Studies have elucidated that EVs from glioblastoma (GBM) under hypoxic conditions produce growth factors and cytokines which in turn activate directed pericyte movement and PI3K/AKT signaling and induce angiogenesis (Matarredona, 2020). Such EVs carry molecules like VEGF-A which straightaway promote angiogenesis (Are, 2016; Kalluri, 2016; Tomasetti et al., 2017). ECS have also shown enhanced sprouting and bifurcation of vessels after being delivered the components of the Notch pathway *via* exosome (Whiteside, 2017).

Exosomes from cancer cells have also been noted to bring about changes in fibroblasts *via* the TGFβ/Smad pathway. Cancer-Associated Fibroblasts (CAFs) can augment or inhibit tumor growth driven by tumor-derived exosomes (Whiteside, 2017; Sun Z. et al., 2018). Another study showed that the transfer of CRE mRNA to normal cells *via* EVs had pronounced immunosuppressive character (Are, 2016). The oncogenic potential of tumor cells is retained by the removal of tumor suppressor miRNAs *via* exosomes. Ras-MEK network is involved in maintaining the RNA-Induced Silencing Complex (RISC) which in turn leads to the release of miRNA in exosomes (Rachel et al., 2017). The uptake and attachment of exosomes in target cells are mediated by protein interactions *via* several adhesion molecules like integrins and ICAMs whereas heparin sulphate proteoglycans, carbohydrate/lectin receptors, T-cell immunoglobinmucin-binding phosphatidylserines, *etc*. are crucial for their entry inside the cells (Tomasetti et al., 2017). In another tumor microenvironment, the exosomes of prostate cancer cells were rich in H-Ras and K-Ras signaling mechanisms. They contained miRNAs and Rab proteins, which led to aggressive tumors in the recipient target cells. Likewise, cancer-cell derived exosomes having copious presence of Ras and other kinases in the MAP kinase pathway, phosphorylated EGFR, and other growth factors led to an increased longevity of tumor monocytes. It has further been demonstrated that the capability of tumor cells to manipulate healthy distant cells involving exosomes is *via* a mechanism needing Rho GTPase effectors Rac1/PAK2 which possibly is the basis of metastatic spread of tumors (Rachel et al., 2017). The exosomal signaling is not only dependent on growth factors and cytokines but several metabolites as well, which include lactate, proteins, ketone bodies, *etc*. (Whiteside, 2017).

As a new and potent method to combat cancer, targeting the precise signaling pathway of exosomes is an exceedingly promising and emergent aspect of cancer therapy (Sun Z. et al., 2018). Refined manipulation of exosomes, for instance by protein fused to a ligand that allows the targeted delivery of exosomes to neuronal cells is one such successful attempt at modifying and channeling the exosomal signaling mechanism for a noble use of therapeutics (Kalluri, 2016).

## Role of exosomes in cancer: Biomarkers

The diverse cargo of exosomes consisting of circulating RNAs, proteins with membrane and cell functions are proving to be recent research tools as potential biomarkers. A number of recent studies are looking into the insights of exosomal release as indicative of patho-physiological conditions and not merely riddance of undesirable components (Lin et al., 2015). The results have been suggestive of superior rates of sensitivity and specificity involving diagnosis of various metabolic and infectious diseases and cancer tumors by exosomal cargo biomarkers (Lin et al., 2015; Wong and Chen, 2019). This ability is credited to potentiate and better coordination of intercellular communication exhibited by exosomes, amongst a deck of other responses such as oncogenic growth, tumor progression and signaling pathways (Ruivo et al., 2017; Batista and Melo, 2019; Mathew et al., 2020). Challenges and shortcomings of existing biomarkers such as invasive and predictive nature, limited responsiveness and incompetency of genomic biomarkers in efficiently determining adaptive immune responses (Conway et al., 2018; Mathew et al., 2020); have paved way for development of novel diagnostic tools: the exosomal engineered biomarkers. Islet autoantibodies, like GAD65, IA-2 also known as ICA512, accurately predict development of type 1 diabetes mellitus; likely reduce with the disease progression and the antibodies gets exhausted as soon as insulin therapy is initiated (Towns and Pietropaolo, 2011; Garcia-Contreras et al., 2017). The cytokine stimulated ß-cells releasing EXOs are hence being utilized for TID diagnosis involving analysis of specific proteins and RNAs preceding isolation by using a surface marker (Palmisano et al., 2012; Garcia-Contreras et al., 2017).

In 2009, a study reported elevated levels of CD63^+^ (scaffolding membrane protein) exosomes in plasma isolated from melanoma patients; qualifying as a tumor-associated marker based on western blot and flow-cytometric analysis (Logozzi et al., 2009; Lin et al., 2015). The tetraspanin family member was also found helpful in carrying out comparison of various human cancers; when quantification data showed lower levels of CD63 in exosomes derived from non-cancer cells. In addition, they also proposed reliability of CD9 and CD81 as marker proteins based on the fact that both of them were profoundly found in all the (four prostate and five breast) cell lines, that they utilized (Yoshioka et al., 2013; Lin et al., 2015). Several other exosomal proteins have also found their place as suitable prognostic tools in clinical and disease pathologies.

Findings of Taylor et al. regarding exosomal associated eight miRNAs (miR-21, -141, -200a, -200b, -200c, -203, -205, -214) are suggestive of the applications in diseases like ovarian cancer as substitute biomarkers; involving plasma biofluid and are reported to overcome the invasive isolation and profiling of biopsy samples (Taylor and Gercel-Taylor, 2008; Lin et al., 2015). In this regard, cell culture medium biofluids; have found predictive use in metastatic gastric cancers. Let-7 family miRNAs enriched extracellular fractions indicated by signal intensity data, confirming the possibility of selective secretion, and consequently revealed a new disease marker (Ohshima et al., 2010; Lin et al., 2015). Thus, exosomal nucleic acids also possess great potential as biomarkers for cancer diagnosis.

Such expositions by researchers are satisfactory and are coaxing others to take this descriptive nature of the published accounts to a further expanded diagnostic setting, particularly development of ideal biomarkers (Tables 1, 2).

**TABLE 1:** Exosomal proteins as biomarkers for diagnosing cancer from serum and plasma.

Type of tumor	Protein biomarker(s)	Prospective use(s)
Lung cancer	NY-ESO-1 (Sandfeld-Paulsen et al., 2016a)	Prognosis
	EGFR, KRAS, Claudins, RAB-family proteins (Clark et al., 2016)	Diagnosis
	CD151, CD171, tetraspanin (Sandfeld-Paulsen et al., 2016b)	Diagnosis
Breast cancer	Her2 (Ciravolo et al., 2012)	Diagnosis
	Fibronectin (Moon et al., 2016a)	Early Diagnosis
	Glypican-1 (Melo et al., 2015)	Diagnosis
	Breast cancer resistance protein (BCPR) (Chen et al., 2015)	Prognosis
	Periostin (Vardaki et al., 2016)	Diagnosis
	Del-1 (Moon et al., 2016b)	Prognosis
Colorectal cancer	CD147 (Tian et al., 2018)	Diagnosis
	CEA (Huber et al., 2005)	Diagnosis
	Hsp60 (Campanella et al., 2015)	Diagnosis/Therapy
	TSAP6/CEA (Silva et al., 2012)	Prognosis/Diagnosis
	Copine III (Sun Z. et al., 2018)	Diagnosis
Prostate cancer	ephrinA2 (Zhu et al., 2018)	Diagnosis
	surviving (Khan et al., 2012)	Diagnosis
	PTEN (Gabriel et al., 2013)	Diagnosis
	PSA (Logozzi et al., 2017)	Early Diagnosis
	CA IX (Logozzi et al., 2020)	Diagnosis
Pancreatic cancer	CD44v6, CD104, Tspan 8, EpCAM (Madhavan et al., 2015)	Prognosis/Diagnosis
Gastric cancer	TRIM3 (Fu H. et al., 2018)	Diagnosis
	GKN1 (Yoon et al., 2018)	Prognosis/Diagnosis
	HER-2neu, EMMPRIN, MAGE-1, C-MET (Baran et al., 2010)	Diagnosis
Ovarian cancer	TGF-β1, MAGE3/6 (Szajnik et al., 2013)	Prognosis/Diagnosis/Therapy Tracking
	EpCAM, CD24, CA-125 (An et al., 2015)	Diagnosis
Melanoma of skin	Hsp70, Hsp90 (Peinado et al., 2012)	Prognosis
	Caveolin-1 (Logozzi et al., 2009)	Diagnosis
	(phospho)Met (Peinado et al., 2012)	Diagnosis
Hematological malignancies	CD9, CD13, CD19, CD30, CD38, CD63 (Caivano et al., 2015)	Diagnosis

**TABLE 2:** Exosomal nucleic acids as biomarkers for diagnosing Cancer from serum and plasma.

Type of tumor	Nucleic acid biomarker(s)	Prospective use(s)
Lung cancer	miR-151a-5p, miR-30a-3p, miR-100, miR-629 (Cazzoli et al., 2013)	Early Diagnosis
	let-7-g-5p, miR-24-3p, miR-223-3p, miR-7-5p, miR-424-5p (Rodríguez et al., 2014)	Diagnosis
Breast cancer	miR-101, miR-30a-3p, miR-373 (Eichelser et al., 2014)	Diagnosis
	miR-1246, miR-21 (Hannafon et al., 2016)	Diagnosis
Colorectal cancer	miR-19 (Matsumura et al., 2015)	Prognosis
	miR-21 (Tsukamoto et al., 2017)	Prognosis
	miR-221 (Liu et al., 2018)	Prognosis
	miR-4772-3p (Liu et al., 2016)	Prognosis for recurrent stages II and III
	let-7a, miR-1246, miR-150, miR23a (Ogata-Kawata et al., 2014)	Early Diagnosis
Prostate cancer	miR-141 (Li et al., 2016)	Diagnosis
	miR-1290, miR-375 (Huang et al., 2015)	Prognosis
Pancreatic cancer	miR-17-5p, miR-21 (Que et al., 2013)	Prognosis/Diagnosis
	circ-IARS (RNA) (Li et al., 2018)	Diagnosis
	miR-1246, miR-4644, miR-3976, miR-4306 (Madhavan et al., 2015)	Diagnosis
	miR-451a (Takahasi et al., 2018)	Prognosis
	miR-191, miR-21, miR451a (Goto et al., 2018)	Diagnosis
Gastric cancer	miR-423-5p (Yang et al., 2018)	Prognosis/Diagnosis
	LncRNA HOTTIP (Zhao et al., 2018)	Diagnosis
	Circ-KIAA1244 (Tang et al., 2018)	Diagnosis
Ovarian cancer	miR-373, miR-200a, miR-200b, miR-200c (Meng et al., 2016)	Prognosis/Diagnosis
	miR-21, miR-214, miR-203, miR-205, miR-141 (Taylor and Gercel-Taylor, 2008)	Prognosis/Early Diagnosis
	miR-21, miR-100, miR-200b, miR-320 (Pan et al., 2018)	Diagnosis
Hepatocellular carcinoma	miR-718 (Sugimachi et al., 2015)	Prognosis/Diagnosis/Recurrence
	miR-18a, miR-221, miR-222, miR-224 (Sohn et al., 2015)	Diagnosis
	LINC00161 (Xu et al., 2018)	Diagnosis

### Lung cancer

Multiple genes and proteins have been identified in lung cancer cells and tissues that can serve as exosomal biomarkers for lung cancer. The most prominent ones are the proteins (ZEB1, TRAF4, TGF-β, *etc*.) involved in lung cancer metastasis by EMT, proteins (PD-L1, EGFR, TLR7, TLR8) involved in inhibiting the immune system, and several Wnt proteins (Wnt5b, Wnt3a) and Interleukins (IL-6, IL-8, IL-10) that allow invasion and proliferation of the tumor (Jiang et al., 2021). A number of miRNAs that are an integral part of the exosomal cargo of the lung cancer cells have also been noted. They include miR-660-p5, miR-29a, miR-21 and miR494 (promote proliferation of lung cancer cells), miR-5100, miR-9, miR-23a (promote metastasis), miR-21, miR-29 (promote angiogenesis), and miR-23a (involved in immunomodulation) (Xu et al., 2021).

### Breast cancer

As seen with other cancers, exosomes derived from breast cancer cells are enriched with certain miNAs that are not abundant in healthy cells. miR-372, miR-101, and miR-373 were found in significantly higher proportions in exosomes from breast cancer cells. Further, these miRNA are also indicative of metastasizing cancer while miR-373 is a marker of the highly aggressive triple-negative phenotype of breast cancer (Joyce et al., 2016). Expression levels of proteins such as ER (estrogen receptor), Ki67 (a marker of proliferation Ki-67), PR (progesterone receptor), and HER2 (member of the epidermal growth factor receptor family which is involved in the regulation of cell growth, survival, and differentiation *via* targeting multiple signal transduction pathways) can serve as important biomarkers for breast cancer prognosis and diagnosis (Jafari et al., 2018).

### Colorectal cancer

Certain nucleic acid molecules and proteins may also serve as diagnostic biomarkers of colorectal cancer (CRC). Among miRNAs, around 7–11 molecules have been identified to be differentially expressed in CRC patients out of which miR-23a, miR-1246, and miR-21 are considered better markers. Several lncRNAs (colorectal neoplasia differentially expressed-h (CRNDE-h), breast cancer anti-estrogen resistance 4 (BCAR4), mRNA keratin-associated protein 5-4 (KRTAP5-4), and mRNA melanoma antigen family A3 (MAGEA3) have also been found in higher amounts in serum exosomes, thus increasing the scope of using them as predictive as well as diagnostic molecules. Upregulation and downregulation of specific proteins may serve as another method to screen CRC patients. Heat shock protein 60 (a chaperonin involved in tumorigenesis), glypican-1, and the transmembrane protein CD147 are increasingly expressed in Colorectal Cancer, hence could be potential candidates for diagnosis (Balacescu et al., 2018; Xiao et al., 2020).

### Pancreatic cancer

Similar to Colorectal Cancer, the cell surface proteoglycan Glyptican1 (GPC1) is the most prominent biomarker of pancreatic cancer. Exosomes enriched in GPC1 are known to positively regulate cancer and thereby serve as the best biomarker for detecting pancreatic cancer (Melo et al., 2015). Further, based on the study of exosomes isolated from pancreatic cancer cell lines and plasma isolated from patients, miRNAs such as miR-196a, miR-1246, miR-191, miR-21, miR-451a, miRNA-483-3p, miR-155, miR-196a, *etc*. are present in ample amount in the pancreatic cancer tumor microenvironment and may be effectively used to diagnose the same (Gabriel et al., 2020).

### Ovarian cancer

Ovarian cancer cells derived exosomes are extracted from either ascites or serum of patients and contain a concoction of specific signature molecules which help in the progress of the tumor. It includes membrane proteins (Alix, TSG 101), Rab proteins, annexin proteins, tetraspanins (CD9, CD82, CD63 and CD81), heat shock proteins (Hsp90, Hsp70), antigens (MHC I and II), Nanog and enzymes (phosphate isomerase, peroxiredoxin, aldehyde reductase, fatty acid synthase), which can be used to potentially identify the malignancy early in itsdevelopment (Feng et al., 2019). Since ovarian cancer is highly lethal yet lacks any early screening test, therefore using exosomalmiRNA biomarkers (miR-100, miR-200b, miR-320, miR-21, miR-362-5p, and miR-1274a *etc*.) for diagnosis and prognosis of ovarian cancer would be of great clinical utility (Yoshida et al., 2020).

### Gastric cancer

*Helicobacter pylori* infection is the most common factor that predisposes a person to develop gastric cancer. Interestingly, studies have found the role of exosomes in *H. pylori* infection and tumorigenesis by transporting the virulence factor CagA (mediator of extragstric disorders) to epithelial cells and mesenchymal-epithelial transition factor (MET) protein to macrophages, thus aiding in disease progression (Tang et al., 2021). Other signature molecules that form a part of the exosomes cargo from GC cells include proteins (UBR2, TRIM3, Apolipoprotein E), miRNAs (miR-423-5p, miR-155-5p, miR-27a, *etc*.), IncRNA (ZFAS1, LINC00152), and circRNA (ciRS-133, circ-KIAA1244) which may be utilized as characteristic biomarker for early diagnosis of gastric cancer (Fu et al., 2019).

## Exosomes as therapeutic targets

### Modification of exosome content

By default, exosomes are generally engineered under the control and governance of various cellular mechanisms; however, an accelerating number of successful researches are presently being done that involve exploring possibilities of exosomal content modification. The biocompatible traits of exosomes, with several appropriate changes, can trigger the steadiness and efficacy of cellular uptake and prove to be an effective step in improving the picture of current therapeutics. The cognizance of this subject matter is to summarize perspective, passive and lively approaches to unique exosome changes, and examples of the transport molecules (Luan et al., 2017).

Exosomal cargos such as nucleic acid components, heat shock proteins, and various ligand molecules, for example, miR-425-3p, TGF- β, miR-100–5p, and Survivin, are found to be effectively involved with various targeted cells such as TGF- β is with NK cells. These modified exosomes, due to the incorporation of desirable components can prove to be an efficient vehicle in dealing with advancing cases of cancers such as AML, Lung cancers, Hepatocellular cancers, *etc*. A platinum-based chemotherapeutic approach was carried out to monitor a Lung cancer model, utilizing exosomal content modification and miR-425 as the cargo component. It was rendered ineffective resulting in a resistive response to NSCLC which was an outcome of autophagy due to AKT1 inhibition. Similarly, NK cell-mediated suppression controlled by tumor-derived vesicles proved to be therapeutic in instances of AML accompanied by the opposition of suppression by interleukin-15. The study outcome in the case of A549 cells involved DDP resistance and was observed in resident cancer cells due to modulation of mTOR expression. In another study, Exosomes purified from HeLa Cervical Carcinoma cells exhibited revised survival rates due to assistance from IAP and HSPs (Refer to the table).

One such critical group of derived exosomes comes beneath the mega-group of Tumor-derived exosomes. There are an array of attractive components that contributes to the usage of tumor-derived exosomes for the transport of therapeutics and vaccines for immunotherapy. A stage 1 medical trial has recently been accomplished on the discharge of tumor exosomes, which had been earlier presumed to undergo tumor specificity *via* antigens equipped for presentation to immune cells and stimulating the immune structures of glioma sufferers to achieve pure and ultimate tumor cells after resection (Thomas Jefferson University, 2000).

For example, for the determination of tumor cells and tumor exosomes in excessive numbers in malignant effusions, it has been established that tumor exosomes convey tumor-related antigens unique to the tumors from which they may be derived, in addition to MHC I molecules. A supply of antigens to dendritic cells by tumor exosomes can result in a T-cellular-mediated immune reaction towards tumor cells (Wolfers et al., 2001). In addition, tumor-focused on selective drug transport involves tumor-derived exosomes and has been proposed as an opportunity due to their unique expression of tetraspanins, which preferentially engage with ligands in specific tissues (Rana et al., 2012). Proteases, including urokinase plasminogen activator, which promotes tumor cellular invasion, and cathepsin D, and adhesion modulators, including vimentin, galectin 3-binding protein, and annexin A1, have additionally been determined in tumor-derived exosomes (Harris et al., 2015); miRNAs and different nucleic acids, that may result in malignant adjustments in target cells, had been identified in tumor cellular exosomes (Melo et al., 2014). An account of exosomal alterations manifested as therapeutic and restorative in specific cancer treatments is enlisted below in Table 3.

**TABLE 3:** An account of exosomal alterations manifested as therapeutic and restorative in specific cancer treatments.

Exosomal cargos	Targeted cell	Cancer model	Study outcome	Reference
miR-425-3p	PC-9 and SPCA1 cells	Lung Cancer	AKT1 inhibition triggers autophagy, conferring resistance to NSCLC; observed as decrease in clinical response to platinum-based chemotherapy	Zhao and Xie, (2019), Yuwen et al. (2019)
miR-100–5p	A549 cells (Rapamycin signaling pathway)	Lung Cancer	DDP resistance was observed in other cancer cells due to modulation of mTOR expression	Qin et al. (2017), Zhao and Xie, (2019)
miR-222	MCF-7/S (Phosphatase and tensin homolog)	Breast Cancer	Exosomes were found to be acting as MDR arbitrators that transferred Adriamycin-and Docetaxel resistance from donor cells to recipient MCF-7 breast cancer cells	Chen et al. (2014), Zhao and Xie, (2019)
miR-122 and miR-32-5p	HepG2 cells (Sensitive HCC cell)	Hepatocellular cancer	Delivery of miR-122 into HepG2 cells followed by its negative regulation expression; aids the sensitivity of HCC cells to chemotherapeutic agents. miR-32-5p facilitates the activation of PI3K/Akt pathway thus providing MDR to sensitive cells.	Lou et al. (2015), Xiao Fu et al. (2018), Zhao and Xie, (2019)
lnc-ROR and lnc-VLDLR	HepG2 or PLC-PRF5 HCC cells	Hepatocellular cancer	Upregulatory response in HCC cells, reduction in chemotherapy-induced cell death and increased expression level of linc-ROR; and ABCG2.	Takahashi et al. (2014a), Takahashi et al. (2014b), Zhao and Xie, (2019)
TGF- β	NK Cells	AML	NK cell mediated suppression mediated by tumor-derived vesicles which proved to be therapeutic in instances of AML and the fact that interleukin-15 can oppose this suppression, was also established	Szczepanski et al. (2011), Whiteside, (2016)
Hsp72	MDSC	Colon CA	Suppression in the activity of the MDSCs *via* activation of Stat3 triggered by TDE-associated Hsp72 in a TLR2/MyD88-dependent manner *via* autocrine production of IL-6. MDSC expansion was triggered by TDSFs following activation of Erk	Chalmin et al. (2010), Zhao and Xie, (2019)
Survivin	Cervical CA cells	Cervical CA	Exosomes purified from HeLa Cervical Carcinoma cells demonstrated increased survival rates due to assistance to IAP and HSPs	Khan et al. (2011), Zhao and Xie, (2019)
αvβ6 Integrin	Prostate CA cells	Prostate CA	EVs were derived from PCa cell lines and human plasma samples, characterized as to contain ds-gDNA fragments which appears to be an able candidate for cancer biomarker; exhibiting certain specific migration mutations	Lázaro-Ibáñez et al. (2014), Zhao and Xie, (2019)
FasL	Activated T cells	Ovarian CA	An increased immune-suppression of T-cell receptor/CD3-zeta followed by T-cell apoptosis due to modified Fas ligand-containing exosomes obtained from ovarian tumors	Taylor et al. (2003), Matsumura et al. (2015)

ABCG2, ATP-binding cassette sub-family G member 2; AML, acute myeloid leukemia; CA, carcinoma; DDP, Cisplatin [cis-diamminedichloroplatinum(II)]; HCC, hepatocellular carcinoma; Hsp, Heat Shock Protein; IAP, Inhibitor of apoptosis; lnc-ROR, Long Non-coding RNA Reprogramming; lnc-VLDLR, Long Non-coding very low density lipoprotein receptor; MDR, Multi-drug resistance; MDSC, myeloid-derived suppressor cells; miR, micro RNA; mTOR, Mammalian target of rapamycin; NSCLC, Non-small cell lung cancer; NK, natural killer cells; TDE, Tumor derived exosomes; TGF-β, transforming growth factor β; TDSF, Tumor derived Suppressor factor.

#### Modification of exosome surface

Exosomal surface proteins (ligands) can be modified to aid targeted drug delivery. It is carried out to image and track them, make them better adapted to the target cells vis-à-vis anchorage and uptake, increase their therapeutic value, and give them other possible advantages over unmodified exosomes (Sandfeld-Paulsen et al., 2016a). This specific modification is beneficial in terms of more effective drug delivery, more retention in circulation and more stability. Furthermore, since it augments the targeted delivery, a lesser quantity of exosomes is required for the same effect, thus reducing the need for high yield from parent cells (Zhang et al., 2020).

These modifications are done through various methods, depending upon the requirement, including genetic engineering of parent cells, nanoparticle technology, hydrophobic cargo loading in the lipid bilayer, fusion with liposomes, *etc*. (Xu et al., 2020). Table 4 summarizes a list of practical changes carried out on exosomal surfaces to assist their involvement in promoting advancements and therapies.

**TABLE 4:** A list of practical changes carried out on exosomal surfaces to assist their involvement in promoting advancements and therapies.

Source of exosome	Molecule involved in expression	Loading and labeling	Consequences and aftermath	Reference
HEK293	GE11 peptide and microRNA Let-7a	Xenolight DiR	Binding of GE11 to EGFR caused the delivery of exosomes to epithelial originated tumors; increase three-fold and assisting in tumor suppressive target delivery	Ohno et al. (2013), Gilligan and Dwyer, (2017)
Murine immature dendritic cells	Membrane protein Lamp2b fused with αγ integrin-specific iRGD peptide	Dox	Delivery of encapsulated Dox with 20% efficacy significantly inhibited tumor growth; demonstrating a prospective approach of modifying exosomes by delivery target of ligand molecules	Yanhua Tian et al. (2014), Gilligan and Dwyer, (2017)
HEK293T cells	IL3-Lamp2B (Lamp2B conjugated with IL3-receptor)	Imatinib or BCR-ABL siRNA	CML infected mice exhibited improved tumor targeting due to exosomal surface modification. IL3-R is overexpressed in CML blasts therefore appearing as a potential receptor to be utilized in drug deliveries; involving cancer cases. Additionally, in cases involving Imatinib group, a slight reduction in tumor growth was observed	Bellavia et al. (2017), Gilligan and Dwyer, (2017)
AuNP coated exosome particles	Lamp2b fusion protein and a neuron-targeted short peptide of RVG	DiI	AuNPs encapsulated with RVG-targeted exosomes, reported elevation in their targeting ability in both *in vitro* as well as *in vivo* blood-brain barrier systems. This could prove beneficial in drug delivery pathway and diagnosis revolving around CNS disorders such as Alzheimer’s disease, Parkinson’s disease and incidents of brain cancers	Khongkow et al., 2019
CD63-GFP-exosomes	GALA Peptide in cytosol	Dextran, Saponin	Combination of cationic lipids and a pH-sensitive fusogenic peptide caused a substantial increase in cellular uptake and cytosolic release of exosomal contents; without any cytotoxic effects	Nakase and Futaki, (2015), Zhang et al. (2020)

AuNPs, Gold nanoparticles; CML, Chronic Myelogenous Leukemia; DiI, 1,1′-dioctadecyl-3,3,3′,3′-tetramethylindocarbocyanine perchlorate dye; DiR, 1,10-dioctadecyltetramethyl indotricarbocyanine Iodide; Dox, Doxorubicin; EGFR, Epidermal Growth Factor Receptor; GFP, Green fluorescent protein; HEK, Human Embryonic Kidney cell line; RVG, Rabies virus glycoprotein.

### The prospects of exosome mimetics

The role of exosomes in multiple cancer types and their potential utility as targeted therapeutics by its alteration or through exosome-mimetics has been widely discussed in this paper. Additionally, since exosomes are functionally involved and produced by almost all types of cells, their application is also omnipresent and is witnessed throughout various ailments. Apart from cancer, exosomes have been studied as ideal vehicles for drug delivery for several neurodegenerative diseases like Alzheimer’s disease and Parkinson’s disease, cardiovascular disorders, musculoskeletal diseases, Kidney ailments, diabetes, *etc*. (Antimisiaris et al., 2018; Jiang et al., 2019). A revolutionary aspect of exosomes is their ability to act as nanocarriers for the delivery of therapeutic agents in brain to treat disorders pertaining to the CNS (Antimisiaris et al., 2018). Neuron-derived exosomes (NDEs) have also been implied to contain distinguishing biomarkers for HIV-associated neurological disorders (HAND) and Alzheimer’s Disease (AD) (Pulliam et al., 2019).

Studies have also been performed to show how circulating EVs might have a functional role in the pathophysiology of several vascular disorders such as Acute Chest Syndrome (ACS) and Sickle Cell Disease (Lapping-Carr et al., 2020). Experimentally, it has been demonstrated that Mesenchymal Stem Cell Derived-Exosomes (MEX) suppresses lung infection by regulating the lung tissue therefore, thereby lies a possible remedial for Pulmonary Arterial Hypertension (Willis et al., 2018). Urinary exosomes as well give an insight into diverse biomarkers that are pointers to different drug-induced kidney toxicities. Furthermore, this aspect can be scaled up after subsequent research for large-scale drug trials (Griffin et al., 2020).

Preclinical research has displayed the ability of MSC-derived exosomes as an alternative form of therapy for Acute Respiratory Distress Syndrome (ARDS). Furthermore, MSC-derived exosomes behave as silencing complexes; hence they can induce some epigenetic changes in the expression of their cellular receptors, eventually leading to the inability of infection of many RNA viruses like Hepatitis-C, Influenza and Coronavirus. This is suggestive of the underlying aptitude of MSC-derived exosomes to treat COVID-19 infection (Gupta et al., 2020).

\Although the role of exosomes as curative agents is highly promising, there are quite a few limitations that need to be tackled first to unleash its extensive utility. The shortcomings of exosomes therapeutics include scanty yield of exosomes from cells, difficulty in loading drugs and engineering the vesicles, potential unwanted effect at non-target sites, systemic dilution before reaching the target site, change in the conformation of membrane protein (thus affecting organotropism), questionable stability of the engineered-vesicles *etc*. (Antimisiaris et al., 2018; Hu et al., 2020). Thus, the use of exosomes-mimetics, which are membrane-coated nanoparticles having the same functional characteristics and efficacy as endogenous exosomes appear more promising for the same therapeutic purpose. Additionally, it can be quickly produced, engineered and loaded with our desired drug in a reproducible and relatively cost-effective manner (Hu et al., 2020).

## Conclusion

This article has comprehensively discussed exosomes and their quintessential involvement in various facets of Cancer Biology. Firstly, there is enough substantial evidence that solidifies the role of exosomes in cell-to-cell communication and signaling and regulation of tumor micro-environment. Secondly, exosomes aid in malignancy of the disease by promoting metastasis and tumor progression. Moreover, they can be characterized to act as biomarkers for efficient diagnostic applications. Thus, these extra-cellular vesicles are multidimensional in terms of the functions that they perform and have thereby emerged as a highly promising niche for cancer therapeutics. It is remarkable to note their efficient, intrinsic targeting potential, biocompatibility, efficacy and physiological stability.

However, numerous aspects, such as purification, administration, standardization and long-term safety effects, require to be studied and monitored. The current studies also lack clinical trials on human models, which are essential to further advancements in this area. Despite their expanded hopes of applications, more advanced and robust technologies are required to isolate surplus exosomes to counter the scanty yield from the current methods. Also, a scalable and economical method for loading drugs/nucleic acids and modifying the surface of exosome mimetics is awaited that also preserves the integrity and innate characteristics of these engineered vesicles.

We have partly unraveled the search for the perfect, fool-proof tool for the cancer treatment, but the quest is yet incomplete until the abovementioned voids are filled. Conclusively, exosomes undeniably carry a plethora of possibilities to revolutionize and significantly optimize Cancer therapeutics and diagnostics. Even, a multitude of research still needs to be performed and analyzed to apply the pre-clinical proof of concept studies to fruition.

## Abbreviations:

ARF, ADP ribosylation factor; LAMP, lysosome-associated membrane protein; MHC, major histocompatibility complex; MUC1, mucin 1; PGK-1, phosphoglycerate kinase 1; TfR, transferrin receptor.
